# Preparation of DNA/Gold Nanoparticle Encapsulated in Calcium Phosphate

**DOI:** 10.1155/2011/647631

**Published:** 2011-06-07

**Authors:** Tomoko Ito, Koyuki Ibe, Tomohiro Uchino, Hiroyuki Ohshima, Makoto Otsuka

**Affiliations:** ^1^Research Institute of Pharmaceutical Sciences, Musashino University, Shinmachi 1-1-20, Nishitokyo-Shi, Tokyo 202-8585, Japan; ^2^Graduate School of Pharmaceutical Sciences, Tokyo University of Science, 2641 Yamazaki, Noda-Shi, Chiba-Ken 278-8510, Japan

## Abstract

Biocompatible DNA/gold nanoparticle complex with a protective calcium phosphate (CaP) coating was prepared by incubating DNA/gold nanoparticle complex coated by hyaluronic acid in SBF (simulated body fluid) with a Ca concentration above 2 mM. The CaP-coated DNA complex was revealed to have high compatibility with cells and resistance against enzymatic degradation. By immersion in acetate buffer (pH 4.5), the CaP capsule released the contained DNA complex. This CaP capsule including a DNA complex is promising as a sustained-release system of DNA complexes for gene therapy.

## 1. Introduction

Gene therapy has been proposed as a novel strategy for the treatment of refractory disease. However, direct injection of naked DNA coding a therapeutic gene generally fails to exhibit a satisfactory therapeutic effect [1, 2]. The low efficiency is due to the negative charge of the DNA molecules, which interferes with the binding of the complex to the cells. Too large DNA molecules also cause poor uptake by cells. DNase, which is present everywhere in the living body, seems to lower the efficiency of gene expression. Therefore, viral vectors have been widely used as carriers to deliver the therapeutic nucleic acids efficiently to the target cells. However, viral vectors have risks such as random recombination and immunogenicity [2]. Thus, safer alternative nonviral vectors such as polycations or cationic lipids have been explored as transfection mediators [2]. The DNA molecules can electrostatically associate with the cationic reagents and form small particles [3]. However, these DNA complexes are usually positively charged, which invites an adverse interaction with blood components or cells [4–6]. Moreover, the therapeutic effect is not satisfactory because of the short duration of gene expression [7].

 Recently, drug delivery systems composed of inorganic nanoparticles, such as silica nanoparticles [8] or gold nanoparticles [9], have been developed. Gold nanoparticles have the advantages of easy preparation and the possibility of chemical modification on the surface [10]. They also have distinctive optical properties, showing strong surface plasmon bands from the visible region to the near-IR region depending on their shape [11, 12]. Absorbed photoenergy is transformed to thermal energy, which stimulates drug release [13]. It should, thus, be possible to prepare an optically responsive DNA release system by binding DNA complexes to gold nanoparticles through thermodegradable bonds.

 However, DNA/gold particle complexes are generally unstable in plasma because of their positive surface charge [14] and show nonspecific side effects with biocomponents as mentioned above. On the other hand, it is known that calcium-phosphate-based compounds, which have similar inorganic components to bone and teeth, are very biocompatible, and have been used as biomaterials, such as artificial bone or teeth [15]. In addition, they are dissolved and absorbed by the acid secreted from osteoclasts [16]. Such biocompatible and biodegradable materials are promising candidates as novel biocompatible and highly durable drug-releasing devices [17].

In this study, we developed novel DNA/gold nanoparticle complexes with protective calcium phosphate (CaP) coating. The effects of the CaP coating on the protection against degradation by DNase and suppression of adverse interactions with cells were investigated.

## 2. Materials and Methods

### 2.1. Materials

Chloroauric acid (HAuCl_4_), sodium borohydride (NaBH_4_), and 2-aminoethanethiol were purchased from Wako Pure Chemical Industries, Ltd. Hyaluronic acid sodium salt (from a microorganism) and YOYO-1 iodide were obtained from Nacalai Tesque, Inc, and Invitrogen Corp, respectively. GFP-coding plasmid DNA (pDNA) with cytomegalovirus promoter was obtained from Clontech Laboratories, Inc. It was amplified in *Escherichia coli* and purified with a QIAGEN Plasmid Mega Kit.

### 2.2. Preparation of DNA/Gold (Au) Nanoparticle Complex

A solution of 0.01% HAuCl_4_ (2 mL) was reduced using 1 *μ*L of 0.38% NaBH_4_ solution to produce Au nanoparticles. Five microliters of 330 *μ*g/mL pDNA aqueous solution was mixed with aminoethanethiol (AET) solution (5.6 mg/mL, 0.5–8 *μ*L; DNA : AET = 1 : 1.8, 3.6, 7.2, 14.4, and 28.7 (w/w)). After 10 min, 60 *μ*L of Au suspension prepared as described above was added to the DNA/AET solution.

### 2.3. Preparation of DNA/Au Encapsulated by Calcium Phosphate

HA aqueous solution (0.5 *μ*L–20 *μ*L, 4.8 mg/mL) was added to 67 *μ*L of pDNA/AET/Au suspension (pDNA : AET = 1 : 7.2 (w/w)). After stirring at room temperature for 30 min, 1.5 times concentrated simulated body fluid (SBF) [18] was added to the pDNA/AET/Au/HA suspension at a ratio of pDNA : AET : HA = 1 : 7.2 : 23.3 (w/w). The mixture was stirred at 37°C for 24 h.

### 2.4. Electrophoresis

A suspension of pDNA/AET/Au/HA with 1.5 SBF was mixed with an equal volume of 30% NaCl solution and incubated for 24 hours at 37°C to dissociate the DNA complex. The DNA complex was then diluted with pure water to adjust DNA concentration ([DNA] = 2 *μ*g/mL), and dissociation of DNA complex was evaluated by agarose gel electrophoresis ([agarose gel] = 1%).

### 2.5. Measurement of *ζ*-Potential and Size

The sizes of Au nanoparticles and DNA/AET/Au/HA complex encapsulated in calcium phosphate were measured by a dynamic light scattering method (DLS) with a particle analyzer (Malvern Zetasizer Nano ZS). DNA/AET/Au complex or DNA/AET/Au/HA complex suspension was diluted with water to 1 mL, and *ζ*-potential was measured using the same particle analyzer.

### 2.6. SEM-EDS Analysis

DNA/AET/Au/HA encapsulated by calcium phosphate was dropped onto adhesive carbon tape and vacuum-dried overnight. The surface was evaluated by SEM-EDS (JSM-7600F, JEOL Ltd., Japan) operated at 5 kV.

### 2.7. Cytotoxicity

Cytotoxicity of DNA/AET/Au/HA encapsulated by calcium phosphate was evaluated by WST-1 assay as follows: MLC-6 cells, an osteoclast-like cell line derived from a mouse, were seeded onto 24-well plates at 9 × 10^3^ cells per well and cultured for 2 days in McCoy 5A media supplemented with 20% fetal bovine serum (FBS). The primary growth medium was then replaced with 500 *μ*L of fresh McCoy 5A with FBS. DNA/AET/Au/HA encapsulated by calcium phosphate was added to the cells (1.65 *μ*g of plasmid per well). After incubation for 4 hours at 37°C, 500 *μ*L of fresh medium was added to each well. After an additional 20 h of incubation at 37°C, the cells were assayed with Premix WST-1 Cell Proliferation Assay System (Takara Bio Inc.).

### 2.8. Cellular Uptake of the Particles

Plasmid DNA was fluorescently labeled with YOYO-1 at a YOYO-1/nucleotide ratio of 0.1. DNA/AET/Au/HA complex was then made of the fluorescent DNA and mixed with 1.5 SBF to be encapsulated by calcium phosphate (final Ca = 2.6 mM). It was added to the cells (1.65 *μ*g of plasmid per well). After incubation for 4 hours at 37°C, 500 *μ*L of fresh medium was added to each well. After an additional 20 h of incubation at 37°C, the cells were observed by a fluorescence microscopy. 

### 2.9. Enzymatic Degradation of DNA

The protective effect against the enzymatic degradation of DNA by encapsulation with calcium phosphate was evaluated using Hind III (Takara Bio Inc.) as follows: Hind III (0.5 unit) was added to the DNA/AET/Au/HA encapsulated by calcium phosphate suspension (DNA = 190 ng) in accordance with the instructions for the reagent. The degradation of DNA was evaluated by agarose gel electrophoresis ([agarose gel] = 1%).

### 2.10. Statistical Analysis

Significant differences between two independent groups were examined by Student's *t*-test. One-way analysis of variance (ANOVA) was used to determine significant differences among six groups.

## 3. Results and Discussion

### 3.1. Formation of DNA/AET/Au/HA Complex Encapsulated in Calcium Phosphate

**Figure 1:**
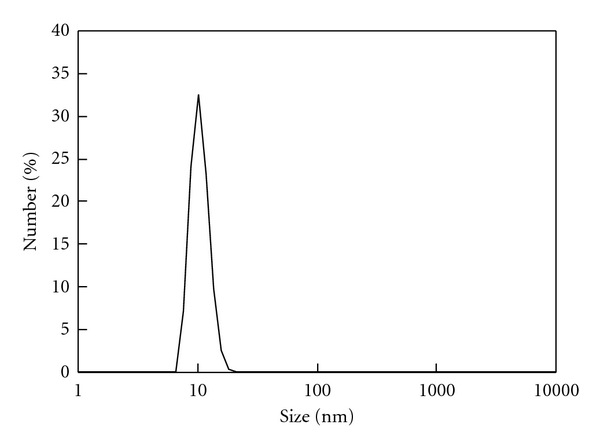
Size distribution profile of the gold nanoparticles.

**Figure 2:**
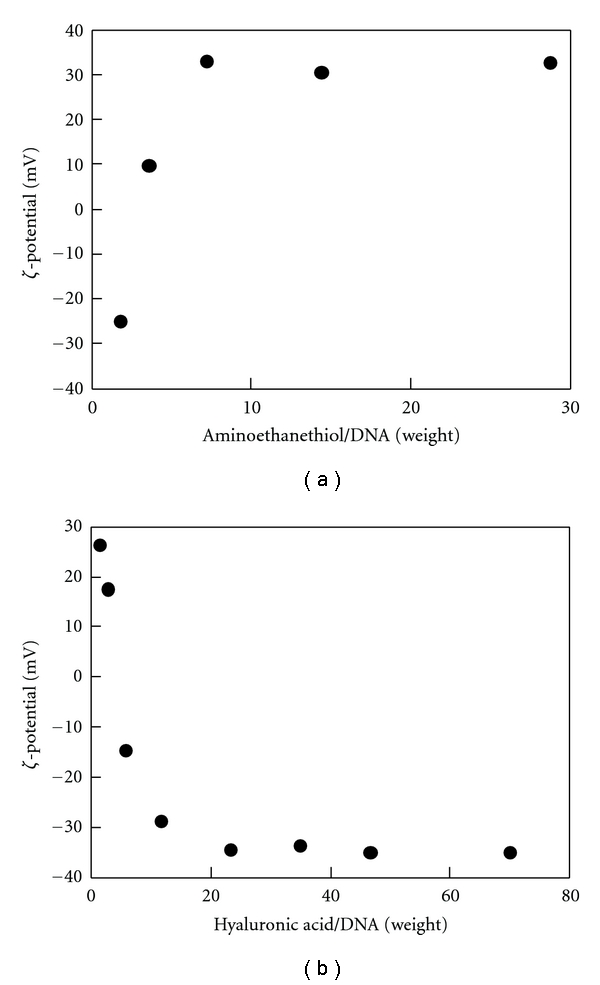
*ζ*-potential of the complex particles composed of (a) DNA, AET, and Au; (b) DNA, AET, Au, and HA.

Small gold nanoparticles were readily obtained by reduction of HAuCl_4_ by NaBH_4_. As shown in Figure 1, gold nanoparticles have a relatively narrow distribution in size with PdI = 0.834. Their number-average size was 10.5 nm with the standard deviation of 1.90 nm. It is known that thiol groups bind to gold nanoparticles [10]. AET was added to positively charge the gold nanoparticle surface. To decide on a suitable ratio of AET to DNA, various volumes of AET solution were premixed with DNA solution and then added to Au suspension. With increasing AET/DNA ratio, *ζ*-potential of the DNA/AET/Au complex increased and was saturated at 33 mV at AET/DNA = 7.2 (in weight) (Figure 2(a)). This ratio, where the highest potential was obtained with the minimal amount of AET, was employed in the following experiments.

DNA/AET/Au complex was then encapsulated in a CaP membrane using SBF. An SBF has a similar inorganic ion concentration to that of human blood plasma and is supersaturated against hydroxyapatite (Ca ion = 2.5 mM). In this study, 1.5 times concentrated SBF (Ca ion = 3.8 mM, pH 7.25) was used to deposit apatite onto the DNA/gold complex surfaces. An apatite layer is known to be formed on bioactive materials with phosphoric acid or carboxylic acid groups [19]. Hyaluronic acid (HA) was then added to coat the pDNA/AET/Au complex suspension to facilitate the deposition of calcium phosphate on the complex. 

Various amounts of HA were added to DNA/AET/Au complex (DNA : AET = 1 : 7.2 in weight), and *ζ*-potential was measured. In line with the amount of HA, the surface charge of the DNA/AET/Au/HA complex decreased and was saturated at −35 mV at HA/DNA ratio = 23.3 (in weight). This ratio, where the *ζ*-potential reaches the lowest level by minimum HA (Figure 2(b)), was employed in the following experiments.

**Figure 3:**
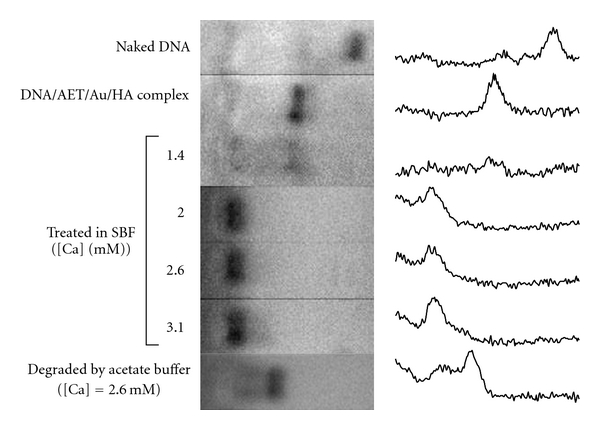
Agarose gel electrophoresis profile of the DNA complexes treated in SBF with various Ca concentrations. Complexes were electrophoresed after dissociation in 15% NaCl. The lowest line represents the result with a DNA complex treated in SBF with [Ca] = 2.6 mM and degraded in an acetate buffer.

DNA/AET/Au complex coated by HA (DNA : AET : HA = 1 : 7.2 : 23.3 in weight) was added to the SBF, and deposition of CaP layer on the surface of DNA/AET/Au/HA complex was attempted. DNA complex suspension was added to 1.5 times concentrated SBF at a final Ca concentration of 1.4, 2.0, 2.6, or 3.1 mM. To examine the deposition of CaP, dissociation behavior of the DNA complex in a concentrated NaCl solution was evaluated. DNA complex immersed in SBF with 1.4 mM Ca ion concentration was dissociated by concentrated NaCl solution and showed bands at similar positions to those of DNA complex without SBF. On the other hand, the DNA complexes immersed in SBF with more than 2.0 mM Ca did not show bands from dissociated DNA molecules. This indicates that CaP could be deposited onto a surface of DNA complex coated by HA by immersion in SBF with more than 2.0 mM Ca and form a stable encapsulated complex (Figure 3). 

Degradation of the CaP capsule in an acidic solution was then examined. An equal volume of pH 4.5 acetate buffer was added to the suspension of DNA/AET/Au/HA complex encapsulated in CaP, which was prepared with 2.6 mM Ca. After stirring at 37°C for 24 h, 30% NaCl solution was added. When it was electrophoresed, a clear band of the dissociated DNA molecule was observed (Figure 3). This shows that the DNA complex was coated with a CaP layer, which could be dissolved in the acidic conditions, and released the DNA complex encapsulated inside.

**Figure 4:**
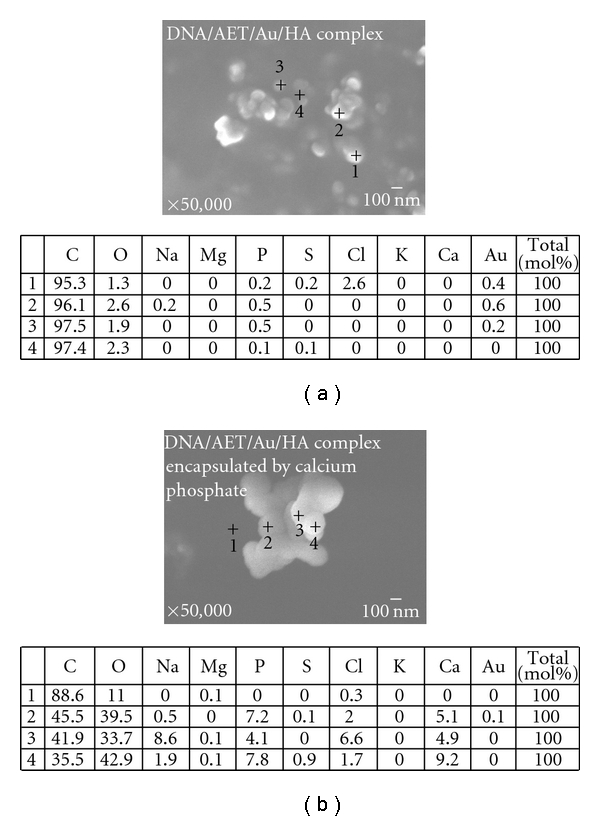
SEM-EDS analysis of (a) DNA/AET/Au/HA complex; (b) DNA/AET/Au/HA complex encapsulated in calcium phosphate (prepared in SBF with [Ca] = 2.6 mM).

**Figure 5:**
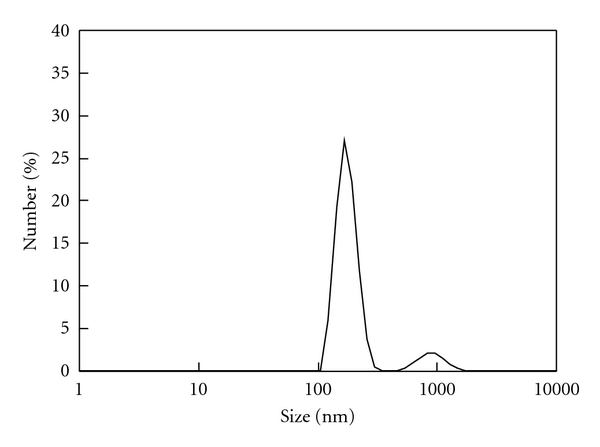
Size distribution profile of the DNA/AET/Au/HA complex encapsulated in calcium phosphate (prepared in SBF with Ca = 2.6 mM).

In SEM-EDS images, differences in surface morphology and composition were observed (Figure 4). Large aggregation of particles with a diameter of ca. 100 nm was seen in the SEM image of DNA/AET/Au/HA complex, and Au was detected in the particle by EDS analyses (Figure 4(a)). On the other hand, the DNA/AET/Au/HA complex encapsulated in CaP was an aggregation of particles of 200 nm in diameter. Calcium and phosphorus were detected in it instead of Au (Figure 4(b)). It was confirmed that the DNA/AET/Au complex coated with HA could be encapsulated with CaP by immersion in SBF. The number average size of the DNA/AET/Au/HA complex encapsulated by CaP suspended in water was 175 nm with the standard deviation of 33.4 nm (Figure 5). The large aggregation of the encapsulated complex would be formed through the drying procedure for SEM observation.

### 3.2. Cytotoxicity of DNA Complex Encapsulated in Calcium Phosphate

**Figure 6:**
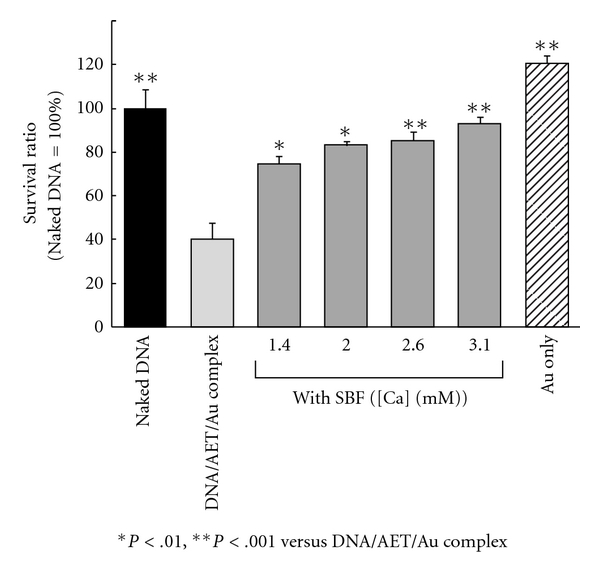
Cytotoxicity of Au nanoparticle and the DNA complexes with or without calcium phosphate envelope.

Figure 6 shows the cytotoxicity of the DNA/AET/Au/HA complex and the encapsulated particles. Judging from the WST-1 assay, DNA/AET/Au complex showed apparent toxicity and only 40% of the cells survived, while Au itself showed much less toxicity. This was due to the cationic surface of the DNA/AET/Au complex. On the other hand, DNA complex encapsulated by CaP showed apparently lower toxicity, and more than 80% of the cells were still alive. Encapsulation by the biocompatible apatite appeared to cause diminished toxicity.

### 3.3. Cellular Uptake of the Particles

**Figure 7:**
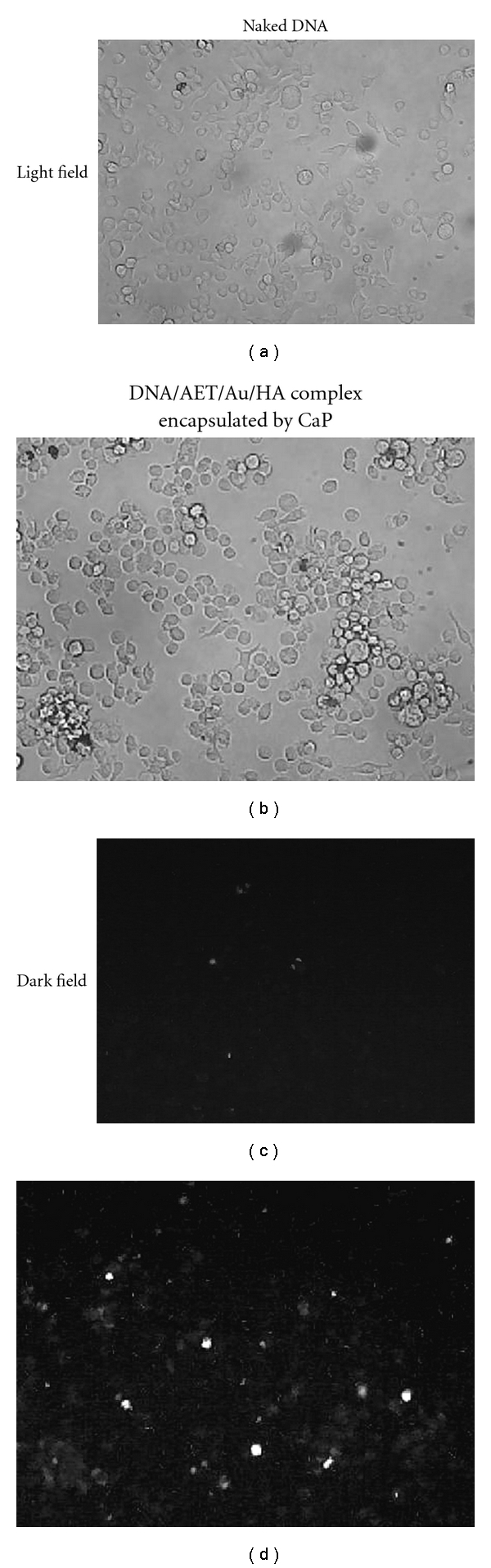
Cellular uptake of fluorescence-labeled naked DNA and its complex with Au encapsulated with CaP.

Plasmid DNA was fluorescently labeled by YOYO-1, complexed with gold, and then packaged by CaP. When they were incubated with MLC-6 cells, the cells became luminescent, while the cells treated with naked DNA/YOYO-1 complex did not show the fluorescence (Figure 7).

### 3.4. Enzymatic Degradation of DNA Complex Encapsulated in Calcium Phosphate

**Figure 8:**
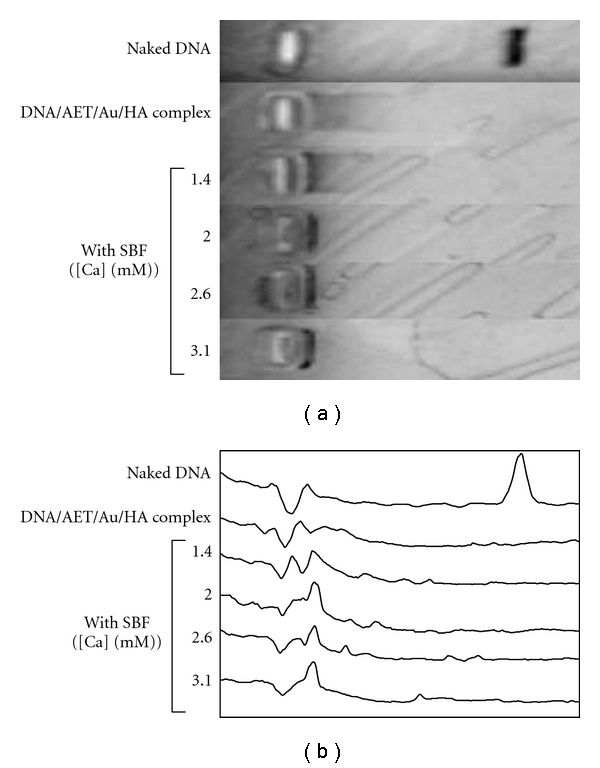
Agarose gel electrophoresis profile of the DNA complex encapsulated in CaP after degradation by Hind III.

Enzymatic degradation behavior of the DNA molecule was evaluated by incubation with Hind III followed by agarose gel electrophoresis. DNA molecule in the DNA/AET/Au/HA complex without CaP was degraded by the enzyme and showed bands of degradation products. A smeared band was observed, unlike for the naked DNA (Figure 8). This was considered to be due to the interaction of the degraded DNA fragments with the cationic polymer. DNA complex not completely encapsulated by CaP, which was prepared in final [Ca] = 1.4 mM, also showed bands of degraded products. On the other hand, DNA complex encapsulated by CaP at more than final [Ca] = 2.0 did not show any DNA fragments (Figure 8). Efficient inhibition of DNA degradation by an enzyme by encapsulation with Cap was confirmed.

CaP-encapsulated DNA/gold nanoparticle has high biocompatibility and resistance against enzymatic degradation and also the releasing property by cellular degradation. It is expected to be a safe and durable nonviral system for gene therapy.

## 4. Conclusion

DNA/gold complex was efficiently included in a CaP capsule by coating the complex with hyaluronic acid followed by immersion in SBF with a Ca concentration above 2 mM. Biocompatibility and resistance against enzymatic degradation were apparently enhanced by the encapsulation with CaP. Incubation of the CaP capsule including DNA complex in an acidic acetate buffer invited the release of DNA complex from the capsule. This shows the high potential of the CaP capsule as an injectable slow-release device, which would release the contained DNA complex by degradation by osteoclasts.
